# PREDICTORS OF LONELINESS INCIDENCE IN CHINESE OLDER ADULTS FROM A LIFE COURSE PERSPECTIVE

**DOI:** 10.1093/geroni/igz038.1345

**Published:** 2019-11-08

**Authors:** Fang Yang, Danan Gu

**Affiliations:** 1 Shanghai University, Shanghai, China; 2 United Nations, New York, United States

## Abstract

Loneliness in older adults is an increasing public health issue. Research on predictors of loneliness incidence using longitudinal data in China is limited. We aim to examine what factors are predictive of loneliness incidence from a life course perspective and whether predictors differ between women and men based on a nationally representative longitudinal dataset in China. A total of 5,043 older adults aged 65 or above from the Chinese Longitudinal Healthy Longevity Survey who were not lonely in the 2008 wave were included in the analysis. Logistic regression models were applied to examine what factors in the 2008 wave predicted loneliness incidence in the 2011 wave. Analyses were also stratified by gender to examine gender differences. Older ages and self-rated poor health increased the odds of loneliness incidence, whereas receiving one or more years of schooling, rural-urban migration, living with family members, having a white-collar job, having a good family economic status, being currently married, having a higher resilience and social support decreased the odds of loneliness incidence. We also found gender differences: socioeconomic factors were significant only for older men, whereas self-rated health, resilience, and social support were significant only for older women. This study offers insights into disentangling the complexity of factors associated with loneliness incidence in Chinese older adults from a life course perspective and from a gendered perspective.

